# Barriers and Facilitators to the Uptake of Online and Telephone Services Targeting Health Risk Behaviours among Vocational Education Students: A Qualitative Study

**DOI:** 10.3390/ijerph18179336

**Published:** 2021-09-03

**Authors:** Prince Atorkey, Christine Paul, John Wiggers, Billie Bonevski, Aimee Mitchell, Flora Tzelepis

**Affiliations:** 1 School of Medicine and Public Health, The University of Newcastle, Callaghan, NSW 2308, Australia; Chris.Paul@newcastle.edu.au (C.P.); John.Wiggers@health.nsw.gov.au (J.W.); Billie.Bonevski@flinders.edu.au (B.B.); Aimee.Mitchell@newcastle.edu.au (A.M.); Flora.Tzelepis@newcastle.edu.au (F.T.); 2 Hunter New England Population Health, Hunter New England Local Health District, Wallsend, NSW 2287, Australia; 3 Hunter Medical Research Institute, Kookabura Circuit, New Lambton Heights, NSW 2305, Australia; 4 College of Medicine and Public Health, Flinders University, Bedford Park, SA 5402, Australia

**Keywords:** barriers, facilitators, uptake, vocational education students, multiple health risk behaviours

## Abstract

Uptake of online and telephone services targeting health behaviours is low among vocational education students and barriers and facilitators are unknown. This study aimed to explore barriers and facilitators to uptake of online and telephone services for smoking, nutrition, alcohol, and physical activity (SNAP) risk behaviours via semi-structured individual telephone interviews with fifteen vocational education students. Two authors independently completed thematic analysis, classified themes according to the COM-B (Capability, Opportunity, Motivation, Behaviour) framework, and discussed disagreements until consensus was reached. Facilitators to uptake of online (e.g., desire to learn something new, cost-free, accessible) and telephone services (e.g., prefer to talk to provider, complements online support) primarily related to capability and opportunity. For telephone services, difficulty understanding accent/language was a capability-related barrier. Opportunity-related barriers for online and telephone services were preference for face-to-face interaction and lack of time, while preference for apps/online programs was a barrier for telephone services. For online and telephone services, not wanting to change SNAP behaviours was a motivation-related barrier and being able to change SNAP risk behaviours themselves was a motivation-related barrier for online services. Barriers and facilitators to online and telephone services are relevant for designing interventions vocational education students are more likely to use.

## 1. Introduction

Both online and telephone support services are effective in assisting adults to quit smoking [1,2,3], increase fruit and vegetable intake [4,5,6], reduce alcohol consumption [7,8], increase physical activity [6,9,10], and modify multiple health risk behaviours [9,11,12]. However, the uptake of such services among the general adult population is low [13,14]. In a UK study, only 0.3% of adult smokers used an internet-based smoking intervention to quit smoking [15]. A recent study among those who were aged 15 years or older in 31 countries reported quitline use that ranged from 0.1% to 4.4% [16]. Use of telephone quitline services by smokers in the US has ranged from 1.6% to 7.3% [17], while in Australia, only 4% of smokers use quitlines every year [14]. Research has also found that less than 1% of adults in New South Wales, Australia, call a telephone service to improve their fruit and vegetable intake and physical activity [18]. Some reasons for low uptake of online and telephone support services include structure of program, mode of delivery, concern about privacy and confidentiality, lack of motivation to use support services, and the impersonal nature of technology-based support services [19,20].

Evidence also suggests that passive engagement which requires individuals to initiate contact with support service providers is a determinant of low uptake of online and telephone services [21,22]. In contrast, proactive engagement, which involves the recruiter initiating contact with the individual, has been found to increase the uptake of such services [21,23,24]. For example, US research has reported that 41% of smokers proactively offered quitline support accepted the offer and received treatment [24], while an Australian study found that 23% of adults accepted proactively offered telephone support to improve nutrition, physical activity, and lose weight [25]. In a study conducted in the Netherlands, more than half (54%) of participants agreed to use an online intervention when proactively offered [26].

A systematic review has reported that a substantial proportion of vocational education students engage in multiple health risk behaviours such as smoking tobacco, poor nutrition (i.e., inadequate fruit and vegetable intake), alcohol use, and physical inactivity (SNAP), highlighting the importance of understanding uptake of support services to modify these behaviours in this population [27]. Vocational education students study courses such as carpentry, floristry, plumbing, automotive engineering, hairdressing, and commercial cooking at vocational education institutions such as trade schools, technical schools, community colleges, and colleges of further education [28]. Despite the potential of proactive recruitment to engage more users, a previous study that examined the uptake of proactively offered online and telephone support services targeting SNAP risk behaviours reported low uptake of these services among vocational education students [29]. In this study, participants who did not meet the Australian health guidelines for smoking tobacco, fruit intake, vegetable intake, alcohol consumption, and physical activity were proactively offered onscreen via a computer tablet online and telephone support services they could use to modify their health risk behaviours [29]. The online and telephone services were QuitCoach and Quitline for smoking, Healthy Eating Quiz and Get Healthy Information and Coaching Service (GHICS), for fruit and vegetable consumption, Tertiary Health Research Intervention Via Email (THRIVE) and Alcohol Drug Information Service (ADIS) for alcohol, and 10,000 steps and Get Healthy Information and Coaching Service for physical activity. Participants who were interested in using the online services were asked to provide their phone number and email address and hyperlinks of the online programs were sent to them. Participants who signed up for telephone services provided their contact details and home and/or mobile phone number and a referral was made to the Quitline and Get Healthy Information and Coaching Service, and these telephone service providers initiated contact directly with participants and provided support. For ADIS, participants provided their contact details and were e-mailed or sent a text with the ADIS phone number to contact. This study found that vocational education students’ uptake of proactively offered online support services ranged from 5.5–14.3% across SNAP risk behaviours while uptake of proactively offered telephone services was between 0.9–7.0% across these risk behaviours [29]. Uptake of support services targeting nutrition and physical activity (12.7–14.5%) appeared higher than that of services for smoking and alcohol (5.5–6.8%) [29]. In relation to modifying multiple SNAP risk behaviours, only 5.8% of vocational education students signed up for online or telephone support to modify two or more health risk behaviours [29].

The barriers and facilitators to taking up an offer of online and telephone services to target health risk behaviours among vocational education students are unknown. To the best of our knowledge, no previous study has explored barriers and facilitators to vocational education students signing up for proactively offered online and telephone support services targeting SNAP risk behaviours. In relation to the general population, an Australian study identified the following barriers to adult smokers not taking up proactively offered support services to assist them to quit smoking: an ability to quit smoking without any assistance, belief that support services would not help them quit smoking, lack of time to use the service, preference for face-to-face support, dislike of technology, and dislike of telephone calls [30]. In another study that proactively offered a telephone service to improve nutrition, physical activity, and to lose weight, participants who did not accept such support identified the following barriers to use: no desire to change, ability to change without any help, belief that the service would not be helpful, time constraints, preference for face-to-face support, dislike of phone calls, and dislike of technology [23].

The COM-B model is a theoretical model for understanding factors that influence behaviour [31], such as the uptake of online and telephone services targeting SNAP risk behaviours. Behaviour change is defined according to the COM-B model as being dependent on three elements: capability (C), opportunity (O), and motivation (M) [31]. Capability refers to a person’s capacity (i.e., psychological or physical) required to perform a behaviour or an activity [31]. Opportunity (i.e., physical or social opportunity) refers to all factors external to an individual that enables a behaviour [31]. Motivation (i.e., reflective or automatic processes) refers to all brain processes that influence the energy and direction of a behaviour [31].

Proactively offering online and telephone services targeting SNAP risk behaviours has the potential to increase the uptake of such services [24,25,26]. However, there has been low uptake of proactively offered online and telephone services targeting SNAP risk behaviours among vocational education students [29]. Understanding the barriers and facilitators to the uptake of online and telephone services targeting SNAP risk behaviours is needed to design effective interventions that vocational education students are more likely to use. Given the lack of existing data among vocational education students regarding the barriers and facilitators to the uptake of online and telephone support services, research is needed to address this evidence gap. This study aimed to qualitatively explore vocational education students’ barriers and facilitators to the uptake of proactively offered online and telephone services for SNAP risk behaviours.

## 2. Materials and Methods

### 2.1. Study Design

A qualitative study comprised of individual in-depth telephone interviews conducted from April 2020 to February 2021 was undertaken in accordance with the Consolidated Criteria for Reporting Qualitative Research (COREQ) guidelines [32]. The University of Newcastle Human Research Ethics Committee granted ethics approval (H-2019-0274).

### 2.2. Participants and Recruitment

Maximum variation sampling was used to recruit participants [33]. Maximum variation sampling is a purposive sampling technique that involves the selection of a broad range of characteristics to maximise the diversity of participants and capture a wide range of perspectives [33]. This study used maximum variation sampling to select a broad range of vocational education students across gender, age, course of study, and area of residence. Students who participated in a previous randomised controlled trial examining the effectiveness of proactively offered online and telephone interventions targeting SNAP risk behaviours [29] and who agreed to be approached for future studies were contacted to capture a variety of experiences related to uptake of services. To be eligible to participate in this study, the following criteria needed to be met: aged 18 years or older, have enrolled in a course at a public Technical and Further Education (TAFE) campus in Australia, have at least one SNAP risk behaviour, and be able to understand English. TAFE is the largest national provider of post-secondary vocational/technical education in Australia [34]. Participants were eligible to participate regardless of their intention to change SNAP risk behaviours because this study wished to capture diverse perspectives about the facilitators and barriers to the uptake of online and telephone services targeting SNAP risk behaviours. Furthermore, research has shown that of participants who accepted a proactively offered telephone service targeting smoking, 72% were not intending to quit smoking within the next 30 days [21]. If this previous study had restricted telephone services to smokers who intended to quit within 30 days, 53.8–65.9% of participants who achieved prolonged abstinence would have missed out on telephone support [35].

Students were e-mailed an information letter describing the research and a consent form in which they were asked to provide a day and time that would be convenient to complete an interview over the telephone or via video communication technology (i.e., Zoom). Those who did not respond to the email were contacted one week later by telephone to ask if they were interested in taking part in the qualitative research. Those who expressed interest were asked to complete the consent form and return it before the interview. Additional approaches used to promote the study included Facebook advertisements, Twitter advertisements, word of mouth, and snowballing. This involved potential participants making contact with the research team via email and they were then e-mailed the information letter and consent form to complete and return.

### 2.3. Data Collection

All interviews were tape recorded and transcribed verbatim. The interviews lasted between 19 to 58 min and were conducted by a trained member (P.A.) of the research team. There was no prior relationship between the interviewer and participants. The interviewer checked the transcripts with the recorded interviews and made notes during the interviews to identify and correct any potential discrepancies in the transcripts. All participants were provided an AUD 25 grocery voucher as reimbursement for their time.

### 2.4. Measures

A semi-structured interview guide informed by the COM-B theoretical model [31] was developed and used to facilitate discussion about barriers and facilitators to the uptake of proactively offered online and telephone support services targeting SNAP risk behaviours. The interview guide was pretested with two individuals prior to the main study. Topics covered in the discussion guide included whether one would use online support and telephone services to change SNAP risk behaviours, and reasons for wanting to sign up or not wanting to sign up for online and telephone support services targeting SNAP risk behaviours. SNAP risk behaviours were measured during the interview by presenting the Australian guidelines for each SNAP behaviour. SNAP risk behaviours were defined as those behaviours not meeting Australian guidelines. Specifically, the interviewer asked: “You might have heard that many Australians don’t meet various health recommendations like not smoking, eating 2 serves of fruit and 5 serves of vegetables each day, drinking no more than 2 standard drinks of alcohol on one day and no more than 4 standard drinks on a single occasion, and doing 150 to 300 min of moderate physical activity or 75 to 150 min of vigorous physical activity each week. Can you think for a moment about which of these recommendations you struggle to meet?”

During the interview, a description of the online and telephone services relevant to each participant’s SNAP risk behaviours was presented and participants were asked about their intended use of these services.

For the online services, the interviewer stated, “There are different online support services available to help people quit smoking, increase their fruit and vegetable consumption, reduce their alcohol consumption, and increase their physical activity.

The QuitCoach is designed to help people quit smoking by providing advice and support on smoking cessation. The QuitCoach creates a personalised quitting plan by asking the individual to answer questions about smoking, their quit plans, motivation, and confidence to quit and past quit attempts. It also provides advice about strategies to help you resist urges to smoke, quitting aids that work, and dealing with nicotine withdrawal.

The Healthy Eating Quiz is designed to improve fruit and vegetable consumption. It examines your intake of a variety of foods. At the end of the quiz, a score is provided with feedback on whether you are eating enough of each type of fruit or vegetable and suggested strategies for increasing the variety of foods you eat.

THRIVE provides advice and feedback about alcohol consumption. It examines your alcohol intake in the last six months and in the last 4 weeks and examines your blood alcohol concentration for your heaviest drinking occasion in the last 4 weeks. THRIVE also estimates the amount of money you spent on alcohol in the last year, provides strategies for reducing alcohol consumption and information about support services.

10,000 Steps is an online program that encourages people to use a pedometer to record their daily step counts on their personal step log, set goal steps, and monitor their progress towards increasing physical activity. Individual walking challenges and virtual walking buddies are also offered, and you can take part in online discussions and access information about physical activity.

Now that you’ve heard about the online programs, would you sign up to use them when offered to help you: i. quit smoking; ii. reduce your alcohol consumption; iii. increase your fruit intake; iv. increase your vegetable intake; and v. increase your physical activity.” Only the online services and options relevant to each participant’s SNAP risk behaviours were presented.

For the telephone services, the interviewer stated, “There are different telephone support services available to help people quit smoking, increase their fruit and vegetable consumption, reduce their alcohol consumption, and increase their physical activity.

The NSW Quitline is designed to help people quit smoking. It offers up to six telephone calls to smokers over one month. The Quitline advisors will talk about dealing with situations that remind you of smoking, provide information on quitting aids that work, and provide support to help you quit.

The Get Healthy Information and Coaching Service is a telephone service that includes information about healthy eating and healthy weight and offers multiple calls to people who use this service. The calls include setting healthy eating goals, strategies for achieving these goals, advice about how to eat a balanced, healthy diet and support to help you improve your eating habits.

The Alcohol Drug Information Service (ADIS) is a confidential and anonymous telephone service that provides information about alcohol use and associated health risks. The ADIS advisors will provide advice about short-term and long-term effects of alcohol, strategies that work for reducing alcohol intake, and information about dealing with stress.

The Get Healthy Information and Coaching Service is a telephone service that includes information about physical activity and healthy weight and offers multiple calls to people who use this service. These calls include setting physical activity goals, tips about how to do enough physical activity each week, and support to help you achieve your physical activity goals.

Having heard about the different telephone support services available for modifying various health risk behaviours, would you sign up to use them when offered to help you: i. quit smoking; ii. reduce your alcohol consumption; iii. increase your fruit intake; iv. increase your vegetable intake; and v. increase your physical activity.” Only the telephone services and options relevant to each participant’s SNAP risk behaviours were presented.

### 2.5. Data Analysis

An inductive approach which allowed for an in-depth exploration of data and theory-driven concepts was employed for the analysis [36]. Thematic analysis of the transcribed interviews was completed using NVIVO (version 12) software. Two members of the research team (P.A. and A.M.) independently read and re-read each transcript and independently assigned codes to the transcribed interviews. O’Connor and Joffe have stated that it is sufficient for two team members to assign codes and reach agreement in qualitative studies [37]. Any discrepancies were discussed between the two members of the research team (P.A. and A.M.) until consensus was reached. Where necessary, a third member of the research team (F.T.) was consulted to resolve disagreements. Codes were categorised into similar themes and presented in terms of the components of the COM-B model [31]. Themes were assigned to the COM-B components by P.A. and F.T. who attended a two-day training course on the use of the COM-B model facilitated by one of the developers of the COM-B model. Any disagreements in the COM-B classifications were discussed between these two members of the research team (P.A. and F.T.) until consensus was reached.

## 3. Results

Fifteen vocational education students participated in an individual telephone interview. The study ceased recruitment after the fifteenth participant was interviewed because data saturation was reached—that is, no new information was emerging during thematic analysis. Our study’s sample size of fifteen participants is consistent with the recommendation that a minimum sample size of twelve is required to reach data saturation in qualitative studies [38,39,40]. Ten participants were recruited from the list of vocational education students who participated in the previous randomised controlled trial, three from Facebook advertisements, and two by word of mouth. Eight participants were female, the mean age was 34.7 years (SD = 13.9), thirteen participants lived in an urban area, and TAFE courses studied were automatic mechanic (*n* = 4), nursing (*n* = 3), English (*n* = 2), floristry (*n* = 2), tertiary preparation (*n* = 1), mental health (*n* = 1), photography and photo imaging (*n* = 1), and community services (*n* = 1). Three participants had one SNAP risk behaviour, seven had two SNAP risk behaviours, one had three SNAP risk behaviours, and four had four SNAP risk behaviours.

### 3.1. Facilitators to Uptake of Online Support Services

#### 3.1.1. Capability-Related Facilitators

##### Desire to Try or Learn Something New

For some participants, learning something new was a reason they would sign up for online support services to change health risk behaviours. This is reflected in the quotes below:

*“I am more than happy to access the information and I am sure I would learn something different from online activities.”*

(Female)

*“If I can learn something then definitely, I can sign up to that one.”*

(Male)

Another participant described how she was already modifying health risk behaviours and was interested in accessing other information via online support services to increase behaviour change.

*“Well, I have already started the changes in my life and I have seen improvements so it can only get better if you access other information as well.”*

(Female)

##### Online Support Complements Telephone Support

Some participants indicated they would sign up for online support to complement telephone support to change SNAP risk behaviours. They would use telephone services but would use online support for further information.

*“I think they both actually complement each other, the online and the telephone. I feel like the online stuff backs up the telephone stuff.”*

(Female)

*“It’s just because the phone call for the fruit and vegetable will probably be like I said one-off or at least periodically, so I don’t feel like I’m choked. Then the online is already ongoing and I can access it when I can. So, yeah.”*

(Male)

#### 3.1.2. Opportunity-Related Facilitators

##### Easily Accessible and Convenient/Flexible

Most participants described online support services as being easily accessible, convenient, and flexible as they could access such services anytime they wanted and at any place.

*“Because, first of all, it’s accessible and anywhere and everywhere.”*

(Male).

*“I think, yeah, if I was to be offered any, then maybe the online, I think it’s probably a bit more convenient online to be able to do it at my own time. So yeah, if I had to choose one, then that is what that would be.”*

(Male)

*“Yes, because at least I can access it any time. It’s more convenient.”*

(Female)

##### Additional Support to Change

The additional support online services would provide to change health risk behaviours was identified as a facilitator to the uptake of online support services. Some participants indicated that online support services would help to maintain behaviour change.

*“I think it is just giving you that support that you need, the offer of support to help you if do tend to slip back into old habits or bad habits or that type of thing.”*

(Female)

For other participants, the online support served as a reminder and provided guidance to engage in health-promoting behaviours.

*“Yeah, I think it’s more about eating habit, because I think I have a problem with that. If I do have to have a program like that telling me that – how much vegetables and fruit I need to consume in a day, it’s like a reminder for me.”*

(Female)

##### Cost-Free to Use and Acceptable Duration

Some participants indicated they would sign up for online support services provided they were free, and the duration was acceptable.

*“I think it would depend on how long it takes and whether there is a cost involved.”*

(Female)

*“Yes, of course, but I think, well, to be honest, anything free of charge, I definitely will sign up for it.”*

(Female)

*“Of course, I will if it’s free. I’m not ready to pay for online.”*

(Female)

#### 3.1.3. Motivation-related facilitators

##### Motivation to Change Health Risk Behaviours

Some participants indicated they would sign up for online support services because the online support services would motivate them to modify their health risk behaviours. Online support services often include motivational components such as goal setting, self-monitoring, and action planning and some participants thought such services would increase and maintain their motivation to modify SNAP risk behaviours.

*“I think that would really motivate me through that, otherwise it is really hard to get the motivation to do it alone.”*

(Female)

*“…. but for me as I told you that I don’t really get motivated to change my behaviours for physical activities and eating healthy foods. If I start following the program, I would end up following it for a long time until I get the results so it would really motivate me to get healthy.”*

(Female)

### 3.2. Facilitators to Uptake of Telephone Support Services

#### 3.2.1. Capability-Related Facilitators

##### Telephone Support Complements Online Support

Some participants indicated they would sign up to use telephone support as it complemented online support services targeting SNAP risk behaviours. Telephone support services were seen as a way to obtain information not captured by online programs and/or to help to understand all the information.

*“Well, I’d do online first…but if I don’t understand something in the online, I can always use the telephone service and get information by speaking. Because sometimes when you speak to people the information is different from just reading the information, Yeah, so sometimes speaking to a person physically or on the telephone because you can hear someone’s voice, it makes a difference.”*

(Female)

*“Probably use the online program, check out different exercises and all that. Then probably use the telephone program as a way of keeping track of it all.”*

(Male)

#### 3.2.2. Opportunity-Related Facilitators

##### Prefer to Talk to Support Provider

Some participants indicated they would sign up for telephone support services because they preferred to talk to someone over the telephone. They thought talking to someone instead of engaging with an online program provided an opportunity to interact and seek further clarification and was more personal and engaging.

*“…because verbal communication, in regard to meals, works best for me. For physical activity, because I can’t really keep track, right. But I think use telephone for just conversation in regards to maybe an effect of a particular strenuous physical activity…But with fruit and vegetables, if someone advises, someone helps you break it down, it’s easier verbally than online.”*

(Male)

*“Again, it’s speaking to somebody. If you feel that it isn’t working as well as you would like it to work, being able to talk to the person about changes that may significantly help and that’s where I think support services are good. Giving you clarification on different foods or different exercises and so forth.”*

(Female)

*“Yeah, you’d actually be talking to a real person as opposed to just online website kind of thing so I guess it’d feel more personal and engaging so I’d probably go for that.”*

(Male)

### 3.3. Barriers to Uptake of Online Support Services

#### 3.3.1. Opportunity-Related Barriers

##### Preference for Face-to-Face or Verbal Communication

Some participants indicated they would not sign up for online support services because they preferred face-to-face interaction or verbal communication. They perceived online support as an impersonal approach that did not provide opportunity for feedback via two-way communication.

*“For me I found myself trying to engage in those things but certainly I need that face-to-face interaction with people. For me, I think and many of the people that I have talked to, an online service wouldn’t work because they need to have a physical body that they’re actually having a face-to-face interaction with and getting feedback from.”*

(Female)

Others felt uncomfortable sharing personal information online and would prefer face-to-face interaction with someone they trusted.

*“I’d probably be more inclined to be face-to-face with someone, rather than receiving online support. That is when it comes to personal things like that, I guess, yeah, and talking to someone that I trust.”*

(Male)

##### Lack of Time

Some participants mentioned lack of time as a barrier to using online services to modify SNAP risk behaviours. Work commitments and other competing demands were reasons for participants’ lack of time.

*“Just time really. Time is hard when you’re working and like you just want to chill out and you don’t really think about anything I guess.”* (Male)

*“Yeah, because sometimes there’s just too many things…when you are signing up with different thing and it just takes time...”* (Male)

#### 3.3.2. Motivation-Related Barriers

##### Do Not Want to Change SNAP Risk Behaviours

Some participants indicated they would not sign up for online support because they did not want to change their SNAP risk behaviours. Participants perceived their health risk behaviours were not problematic and hence did not see a need to change.

*“I don’t need to. I don’t want to because I don’t have a problem, not a big problem I don’t need help in all that.”*

(Female)

*“Yeah, I see how it’s really useful for some people but I don’t really think I have a problem with too much alcohol consumption so I probably wouldn’t end up using it.”*

(Male)

##### Able to Change SNAP Risk Behaviours Themselves

Some participants mentioned they would not sign up for online support services to change SNAP risk behaviours because they perceived they were able to modify these behaviours themselves.

*“Yes, I don’t believe I need that additional support. I believe that I am quite capable of doing it myself. I have the ability to change. I sound like a typical alcoholic, don’t I? I have the ability to change my own behaviours.”*

(Female)

*“No, but I don’t really need help for that. I can do it by myself.”*

(Female)

Others believed they were informed about how to modify SNAP risk behaviours and therefore did not require assistance from online services.

*“Probably not, because I know about what is right to eat, sort of things of what you should be eating. I don’t think I need somebody else to tell me about that. Yeah, it is pretty basic.”*

(Female)

### 3.4. Barriers to Uptake of Telephone Support Services

#### 3.4.1. Capability-Related Barriers

##### Difficulty Understanding Accent or Language

Some participants mentioned difficulty understanding accent and/or language as a barrier to signing up for telephone support services for SNAP risk behaviours.

*“It’s really hard for me to communicate through the phone…you don’t really understand what people are talking about…Language is really restrictive.”*

(Female)

*“You know, trying to get information and you can’t understand what a person is saying. So terrible. You know?”*

(Female)

#### 3.4.2. Opportunity-Related Barriers

##### Preference for Face-to-Face Interaction

Some participants indicated they would not sign up for telephone support services targeting SNAP risk behaviours because they preferred face-to-face interaction. Participants felt that having a telephone conversation denied them the opportunity to respond to visual cues such as facial expressions.

*“To be honest, I wouldn’t sign up because, personally, I don’t like to have this phone call. I like to have more face-to-face interaction, because phone calls, I don’t think it work for me. It’s really hard for me to communicate through the phone… sometimes you have to look at the person facial expression to tell exactly what they mean, and the tone and the voices as well.”*

(Female)

*“Yeah. My preference is for face-to-face.”*

(Female)

For other participants, face-to-face interaction offered the opportunity to be accountable to someone and to discuss strategies in-person for modifying SNAP risk behaviours on a regular basis. Face-to-face interaction was also seen as a way of building a trusting relationship between the client and support service.

*“I can’t think what else there is other than face-to-face. Counselling type of services, I guess, where you’re actually accountable and going and speaking with someone for a period of time…Yeah, those sorts of group support services I think are important and face-to-face, having a face-to-face commitment to somebody on a regular basis to discuss where you’re at, yeah.”*

(Female)

*“Oh yeah, face to face. Yeah, that would be good. Because you actually can sort of trust the person, if you get me.”*

(Male)

Some participants felt face-to-face group interaction would provide support from other people and that talking to someone on the telephone would not offer such peer support.

*“Probably something like meet up or meeting type thing in a way so then I get to meet the teacher or like a leader of the meeting and you can all talk about different things that you can do to try and improve it…I feel like a message from other people would come across to you a lot better than through online or through telephone services.”*

(Male)

##### Preference for Apps or Online Programs

Some participants reported they would not sign up for telephone support services because they preferred apps or online programs for modifying health risk behaviours.

*“But with the eating healthy and stuff, I think a program will be more suitable, because you just need the information, don’t you? You don’t need the … I think it’s tricky, but they’re two very different struggles. I think the eating one is just more about you getting the information about what you can eat and what’s good and what’s not but for eating and that, I think the program will be fine, where I have no person interaction. It’s just information that I’m taking in and absorbing, that’s fine. So yeah.”*

(Female)

Others preferred apps or online programs because they felt they were effective and efficient, and they were uncomfortable talking to people on the telephone.

*“I don’t know. A lot of people these days don’t like talking on the phone. It doesn’t worry me. I think an app would be more effective and a lot better for people because-like a lot of people don’t like talking on phones and whatnot these days. It’s weird but, yeah, a lot of people prefer to use an app. Even with work we see it. A lot of people just because we’ve got an online booking system that instead of ringing up they’ll just use the online booking. Yeah, I think-yeah, an app would probably be more efficient.”*

(Male)

*“Yeah, because with the online one I will just have to follow the tutorials or whatever they are telling me to do. I just would have to follow that. But with the telephone one I think have to give them feedback because I’d be talking to them which I won’t really be comfortable with.”*

(Female)

Others preferred apps or online programs because they were flexible and could be accessed anytime.

*“Online it is, because you can do it anytime. You can do it on flexible time. So for a telephone you would need to have some set time.”*

(Male)

##### Lack of Time

Some participants indicated they would not sign up for telephone services because they did not have the time or did not want to commit time to following a call schedule.

*“Yeah, because of the timing. Because every little time I have, I want to channel it to something, not making calls.”*

(Female)

*“Also, with the telephone services there would be a particular time to talk to them and to provide feedback of what I am doing and all that. I’m not really particular in following the time, timetables and all that stuff.”*

(Female)

#### 3.4.3. Motivation-Related Barriers

##### Do Not Want to Change SNAP Risk Behaviours

Some participants indicated they would not sign up for telephone support services as they did not want to change their health risk behaviours because they did not consider them to be problematic.

*“I guess I don’t feel like I’m at a stage where it’s really, really bad and I do need help.”*

(Male)

*“At the moment, I don’t really think I have a problem.”*

(Male)

## 4. Discussion

This is the first study to explore vocational education students’ barriers and facilitators to the uptake of proactively offered online and telephone support services targeting SNAP risk behaviours. The use of the COM-B model [31] to inform the discussion guide and interpretation of the findings is also an important contribution of this study. Capability-related facilitators to the uptake of online services targeting SNAP risk behaviours involved a desire to try and learn something new and that online services complemented the advice/support received from telephone services. The finding that online support services would help with learning something new is similar to prior research with adolescents [41]. The finding that online services complement advice/support from telephone services is consistent with the study by Lal et al., where participants indicated a preference for support to be offered via different modalities [42]. Providing vocational education students the opportunity to choose the format in which support should be offered may increase their uptake. Opportunity-related facilitators of signing up for online services included that such support was easily accessible convenient/flexible, provided additional support to change, and was cost-free and of acceptable duration to use. These findings are consistent with previous research that found ease of access and/or convenience were reasons adults engaged with digital health interventions [43] and that adults preferred interventions that were brief or shorter in duration [44,45]. A motivation-related facilitator of signing up for online services was motivation to change health risk behaviours. This finding is similar to previous studies that have reported motivation to change health risk behaviours or improve health as a reason for using interventions [46,47,48,49].

In relation to facilitators for the uptake of telephone support services targeting SNAP risk behaviours, a capability-related facilitator reported by vocational education students was that the advice received via telephone support complemented online support services. The option to use telephone support to supplement online programs was seen as providing vocational education students the opportunity to discuss and seek clarification about SNAP risk behaviours via interactive and responsive telephone communication. An opportunity-related facilitator to the uptake of telephone services identified by vocational education students was the preference to talk to a support provider and discuss behaviour change strategies. This finding is consistent with previous studies with adults in the general population where participants preferred verbal communication over online programs to change SNAP risk behaviours [23,50,51,52].

There were a number of barriers identified by vocational education students that were common to both the uptake of online services and telephone services targeting SNAP risk behaviours. Opportunity-related barriers for both online and telephone services were the preference for face-to-face interaction and lack of time, and a motivation-related barrier was not wanting to change SNAP risk behaviours. These findings are consistent with previous research among adults in the general population that indicated a preference for verbal communication or face-to-face interaction to change SNAP risk behaviours [23,50,51,52]. This suggests that a variety of modes for the delivery of support to change SNAP risk behaviours should be offered to vocational education students. Similarly, previous evidence with adult populations has found that lack of time due to busy schedules was a barrier to using support services to change SNAP risk behaviours [23,30,43,53]. Support service providers can consider offering brief interventions that will encourage students to sign up. Vocational education students not wanting to sign up for online and telephone support services because they do not want to change SNAP risk behaviours is consistent with previous Australian research that reported some adults did not want to use proactively offered support to improve physical activity and fruit and vegetable intake [23]. The lack of interest to change SNAP risk behaviours may be because vocational education students do not perceive any immediate consequences of these behaviours. According to the Transtheoretical model, these individuals may be in the precontemplation stage and have no interest in changing SNAP risk behaviours [54]. Emphasising the benefits of modifying SNAP risk behaviours may motivate vocational education students to consider signing up for online and telephone services targeting SNAP risk behaviours [55].

A number of barriers were also mentioned by vocational education students for the uptake of online services only or telephone services only for SNAP risk behaviours. In regard to the uptake of online services, a motivation-related barrier that vocational education students discussed was that they were able to change SNAP risk behaviours themselves and so did not require online support. This is consistent with previous research with vocational education students, which reported that 44% of smokers preferred no support to quit smoking [56], and with adults in the general population who indicated a preference to quit smoking [30], increase fruit and vegetable intake, and physical activity without any support [23]. Vocational education students’ belief that they can change without any support may suggest that education is needed about the effectiveness of evidence-based strategies to modify SNAP risk behaviours. In relation to the uptake of telephone services targeting SNAP risk behaviours, a capability-related barrier identified by vocational education students was difficulty understanding accent or language. This is consistent with a systematic review which reported that language barriers affect patients’ use and satisfaction of health care services [57]. An opportunity-related barrier to the uptake of telephone services was a preference for apps or online programs. This is consistent with a US study with adults that indicated most would use a free app to increase physical activity [58].

Study strengths included that this is the first study to use the COM-B model [31] to explore the barriers and facilitators to uptake of online and telephone services targeting SNAP risk behaviours among vocational education students. The study also had a number of limitations. Firstly, vocational education campuses in Australia offer a wide variety of courses and students from every possible course were not represented, which may limit the generalisability of the findings. Secondly, participants were recruited from vocational education campuses in a high-income country and further research is needed to examine the applicability of these findings to vocational education students in low- and middle-income countries.

Future studies could investigate the extent to which vocational education students who sign up for online and telephone services targeting SNAP risk behaviours engage with these services in terms of duration and number of sessions completed. Future research could also explore the best ways to improve access and promote online and telephone services to make these services more appealing to vocational education students.

## 5. Conclusions

Using the COM-B model [31], this study provides insight into the barriers and facilitators to the uptake of online and telephone support services targeting SNAP risk behaviours among vocational education students. This information is crucial to implementing strategies to overcome barriers to the uptake of online and telephone services such as offering various modes of support services to students, offering brief but effective interventions, and educating students about the benefits of using evidence-based support services to modify SNAP risk behaviours. These findings are important for designing and delivering health behaviour interventions that vocational education students are more likely to use.

## Data Availability

The data presented in this study are not publicly available due to privacy restrictions.
